# Diving Related Changes in the Blood Oxygen Stores of Rehabilitating Harbor Seal Pups (*Phoca vitulina*)

**DOI:** 10.1371/journal.pone.0128930

**Published:** 2015-06-10

**Authors:** Amber Thomas, Kathryn Ono

**Affiliations:** Department of Marine Sciences, University of New England, Biddeford, Maine, United States of America; New York Institute of Technology College of Osteopathic Medicine, UNITED STATES

## Abstract

Harbor seal (*Phoca vitulina*) pups begin diving within hours of birth, stimulating the development of the blood oxygen (O_2_) stores necessary to sustain underwater aerobic metabolism. Since harbor seals experience a brief nursing period, the early-life development of these blood O_2_ stores is necessary for successful post-weaning foraging. If mothers and pups become prematurely separated, the pup may be transported to a wildlife rehabilitation center for care. Previous studies suggest that the shallow pools and lack of diving in rehabilitation facilities may lead to under-developed blood O_2_ stores, but diving behavior during rehabilitation has not been investigated. This study aimed to simultaneously study the diving behaviors and blood O_2_ store development of rehabilitating harbor seal pups. Standard hematology measurements (Hct, Hb, RBC, MCV, MCH, MCHC) were taken to investigate O_2_ storage capacity and pups were equipped with time-depth recorders to investigate natural diving behavior while in rehabilitation. Linear mixed models of the data indicate that all measured blood parameters changed with age; however, when compared to literature values for wild harbor seal pups, rehabilitating pups have smaller red blood cells (RBCs) that can store less hemoglobin (Hb) and subsequently, less O_2_, potentially limiting their diving capabilities. Wild pups completed longer dives at younger ages (maximum reported <25 days of age: 9 min) in previous studies than the captive pups in this study (maximum <25 days of age: 2.86 min). However, captivity may only affect the rate of development, as long duration dives were observed (maximum during rehabilitation: 13.6 min at 89 days of age). Further, this study suggests that there may be a positive relationship between RBC size and the frequency of long duration dives. Thus, rehabilitating harbor seal pups should be encouraged to make frequent, long duration dives to prepare themselves for post-release foraging.

## Introduction

Marine mammals’ ability to successfully locate, capture and consume prey underwater depends largely on their ability to remain submerged for an extended period of time [1]. While underwater, marine mammals have no way of obtaining oxygen from their environment to fuel aerobic metabolic processes; therefore a means to store oxygen collected before diving is necessary for successful foraging [2–4]. Thus, total body oxygen stores can be used to determine the diving potential of an individual [5–10].

Total body oxygen stores refer to the amount of oxygen that can be stored in the lungs, blood and muscle of an individual. Unlike terrestrial mammals, marine mammals store little oxygen in their lungs (5–34% depending on species [11]). Instead, they store oxygen reserves in their blood and muscles, which can be utilized during diving to produce energy as needed [2,12–16]. Pinnipeds (seals, sea lions and walruses) store the largest proportion of oxygen in their blood [17] where it is bound to hemoglobin (Hb) found on red blood cells (RBC). Increased concentrations in RBC (termed hematocrit—Hct) or Hb would result in a higher oxygen carrying capacity in the blood. Adult pinnipeds have the capacity to store significantly more oxygen in their blood than terrestrial mammals of similar size [18]; however, these characteristics are not inborn, but instead develop after birth [15,19].

The mechanism that is hypothesized to drive the development of blood oxygen stores suggests that tissue hypoxia stimulates the release of erythropoietin (Epo) that subsequently stimulates the production of RBC to increase blood oxygen stores [20]. For marine mammals, this hypoxia can be obtained either during “early-life anemia” in which blood plasma increases more quickly than RBC, lowering the oxygen concentration of the blood during the nursing period, or during apnea diving. Thus, in marine mammals, the rate at which the oxygen stores develop in neonates depends on the length of the nursing period [21] and early-life swimming and diving practice [15]. Species that are weaned younger and obtain ample diving practice generally develop large oxygen stores earlier than those that are nursed longer and do not receive much practice [15,21].

Harbor seals (*Phoca vitulina*) are of particular interest in the study of oxygen store development rate because they often begin swimming and diving with their mother hours after birth [22,23]. As such, they have displayed a rapid development of body oxygen stores and diving skills during the nursing period [15,19]. This early life diving behavior may be due to tidal flooding of their pupping grounds, predator avoidance or to reduce the risk of mother/pup separation during maternal foraging bouts [24,25]. Occasionally, nursing pups become separated from their mothers and may then be transported to and cared for in a wildlife rehabilitation center where their swimming is generally limited to shallow pools. This limitation on early life diving could potentially restrict the pups’ access to diving-related apnea and resulting blood oxygen store development [26].

Previous studies on captive unweaned harbor seal pups have shown a decrease in the blood oxygen stores between admission and release, which was presumed to be a result of decreased swimming and diving experience in captivity [27,28]. However, to our knowledge, the relationship between blood oxygen store development during captivity and the diving experience acquired during “normal” rehabilitation has never been investigated. This study aims to: 1) investigate the fine temporal scale blood-oxygen store development of rehabilitating, unweaned harbor seal pups; 2) provide the first documentation of behavioral diving development of rehabilitating harbor seal pups; and 3) determine if the dive-related apnea obtained during rehabilitation is related to blood oxygen storage capacity development.

## Materials and Methods

### Animal Subjects

All harbor seal neonates used in this study were admitted to the Marine Animal Rehabilitation and Conservation program (MARC, Biddeford, Maine, USA) during the spring-summer 2012 pupping season. Only unweaned animals that were admitted due to suspected maternal separation (i.e. unweaned pups without an adult female present that showed no signs of illness) were included in this study, to prevent the possibility of previous foraging-related diving behaviors or illness affecting our results. Upon admission to MARC, all animals underwent a physical examination and a General Health Profile (IDEXX Laboratories, Westbrook, Maine USA) and any necessary fluids, antibiotics or medicines were administered as required. Approximate ages of pups were determined based on the following criteria: presence or absence of an umbilicus, the eruption of teeth, the amount of time they had been observed alone on the beach and overall body condition [15]. Any samples collected from an animal during a period of illness were excluded from the data set to restrict the possibility of any observable changes being attributed to illness instead of development. In total, 21 harbor seal pups ranging from 3–14 days of age at admission were included in this study.

### Blood Collection and Analysis

Blood samples were drawn from the extradural intervertebral sinus [29] into a 3 mL Vacutainer tube and were immediately analyzed on the Oxford Science Forcyte Veterinary Hematology Analyzer. This instrument directly measures hematocrit (Hct), quantity of red blood cells (RBC), hemoglobin (Hb), mean corpuscular volume (MCV), and mean cell hemoglobin concentration (MCHC). Mean cell hemoglobin (MCH) was calculated by the Hematology Analyzer as the average amount of hemoglobin per RBC. Blood was collected only when MARC personnel required blood analysis for healthcare purposes; at least once every 14 days.

### Behavior

Concomitantly with the sampling for blood oxygen stores, 15 of the pups were included in an investigation of their swimming and diving behavior. Newly admitted pups were allowed access to a shallow (10–20 cm) pool for approximately 1 hour every day; a limited number of swimming bouts captured on a security surveillance system were analyzed for the amount of time the animal spent with its nostrils below the water. Breath-hold was chosen to act as a proxy for swimming and diving ability, since these pools were too shallow for diving.

When pups were moved to a room with a deeper pool (1–2 m, Table 1), they were equipped with a dive data recorder (Sensus Ultra Dive Data Recorder by ReefNet, New York, Model: SU-R) that was mounted on a 6 x 8 x 0.6 cm neoprene patch and weighed approximately 50 g. The recorder and mounting apparatus were glued to the dorsal hair of the animal, just posterior of the scapula using NASCO tag cement. The dive recorders began collecting data once the recorder was submerged (1050 mbar of pressure) and collected a data point every 3 seconds until 100 consecutive data points were collected at atmospheric pressure (approximately 1000 mbar) indicating that the animal was no longer in the pool. Each data point included the following parameters: time of day, pressure, and ambient temperature. Pressure in mbar was then converted to depth (m) to analyze the diving behavior of these animals. Eleven dive recorders remained with the same individual throughout the course of the study, while two were deployed on two different individuals.

**Table 1:** Description of the pools available for animals at the Marine Animal Rehabilitation Center [30].

Room ID	Pool Dimensions			
	Length (m)	Width (m)	Depth (m)	Volume (L)
Small Pool 1	0.9 (diameter)		0.1	64
Small Pool 2	1.8	1.2	0.2	430
Wet Isolation	2.6	2.6	1.0	2000
Critical Care	4.9	4.0	1.8	30,600
Communal Rehabilitation 1	5.3	4.0	2.0	39,600
Communal Rehabilitation 2	5.3	4.0	2.0	39,600

All pools had flow-through chlorinated sea water from the Saco River. Generally animals moved through the center from room to room in the order denoted in the table, but some animals were moved to rooms out of order.

Data were uploaded from the Sensus Ultra recorders via a downloader (Sensus Ultra Downloader by ReefNet, New York; Model SU-D) to an Asus (Model K52F) laptop computer. The ReefNet Ultra Software (Sensus Manager) was used to import all data (Table 2). The data were further analyzed using diveMove version 1.3.5 [31], a dive analysis package used within the R environment. Depth recordings had to be at least twice the accuracy of the dive recorder (i.e. ≥ 0.3 m) to be considered a dive (as suggested by Jørgensen et al. [15]). This depth also ensured that a pup could not be breathing at the surface while its tag was recording the behavior as a dive, since the distance between a pup’s nostrils and the placement of the dive tag was less than 0.3 m. For all dives 0.3 m or greater, four variables were calculated per individual animal, per-day using the diveMove software and custom R script [32]: (i) dive duration (s) (mean and maximum); (ii) maximum depth (m); (iii) number of dives; (iv) proportion of “High Intensity Dives” (i.e. dives >150 seconds in duration). This study defines a “High Intensity Dive” as a dive that is likely to cause the PO_2_ of the blood to reach 50% saturation and cause physiological stress. Harbor seal blood has been shown to reach a P_50_ (PO_2_ at which hemoglobin is 50% saturated) at 25.3 mmHg [33] and recent studies with juvenile elephant seals have suggested that the PO_2_ of seal blood reaches a minimum of 25 mmHg during dives of approximately 2.5 min in duration [34]. Due to the larger body size and O_2_ storage capacity in elephant seal pups as compared to harbor seal pups, this is likely to be an over-estimation of the “intensity” of a dive of this duration for harbor seal pups; thus, 2.5 minutes (150 seconds) of continuous breath-hold is considered to be a HID for harbor seal pups. It should be noted that the total number of dive days for all pups recorded (n = 610) is not necessarily continuous. If an animal was considered to be ill, its dive recorder was removed and then re-applied when the animal was considered healthy again (which left a gap in those data). Further, some animals removed the dive recorders from one another, which would leave a gap in the data until the recorder could be re-deployed.

**Table 2:** Individual dive data of rehabilitating harbor seal pups. Admission Age gives the approximated age of the animal when it was admitted to MARC.

Animal ID	Admission Age (days)	Tag Duration (days)	Max DD (sec)	Total Num Dives	Max HID (%)
12–019	5	43	123	15620	0.00
12–020	4	55	215	38621	0.25
12–021	3	68	233	23203	5.70
12–022	3	28	345	74968	0.00
12–023	5	10	156	4594	2.10
12–024	6	79	345	51671	14.4
12–025	5	47	392	20878	5.80
12–029	7	71	311	32910	0.97
12–030	4	29	251	20656	11.35
12–035	7	55	260	34993	20.0
12–038	7	25	413	16839	13.6
12–039	7	25	239	22005	0.95
12–040	10	50	816	27507	14.4
12–041	10	42	657	29428	2.95
12–043	14	8	139	5153	0.00

Tag Duration describes the number of days that a given animal was wearing the Sensus Ultra Dive Recorder. Max DD is the maximum dive duration an animal exhibited during its stay at MARC. The Total Num Dives represents the total number of dives that an animal made while wearing the Sensus Ultra Dive Recorder. The Max HID represents the maximum percentage of High Intensity Dives (dives > 150 seconds in duration) on a given day.

All dive recorders were removed from pups before they were released back into the wild due to our inability to retrieve the tags after release without geo-locator capability. Though this study was unable to follow rehabilitating harbor seal pups through rehabilitation and after-release, observing the rate of change in blood oxygen stores and diving behaviors in the same animals pre and post-release would provide valuable information and should be investigated in the future.

### Statistics

To account for any errors associated with repeated measures and unbalanced data sets due to varying amounts of time in captivity among the pups, linear mixed effects models were used to analyze all hematological and diving parameters [35]. In all models, ‘individual animal’ was used as a random factor. Hematological parameters were analyzed by using measured values (Hct, Hb, RBC, MCV, MCH, MCHC) as the response variable, and age and sex as the explanatory variables. Due to the complex nature of age-related changes, polynomial effects of age were also tested. Diving parameters were analyzed using recorded values (Maximum Dive Duration—Max DD, Mean Dive Duration—Mean DD, Number of Dives made per day—Num. Dives and the percentage of daily High Intensity Dives—HID) as the response variables and age, sex, and available pool depth as explanatory variables. Maximum diving abilities were used in this study because they indicate an animals’ absolute diving ability [8]. To determine if there were any relationships between hematological measurements and diving parameters, a Pearson product-moment correlation test was conducted between hematological measurements and diving measurements. Subsequently, linear mixed effects models were used to determine if a more complex relationship between the measured hematological measurements and the diving parameters existed: the measured hematological measurements were used as the response variables, and age, sex, available pool depth, Max DD and HID were used as the explanatory variables. Model estimates and fitting were obtained using the lme4 package ([36], version 0.999–0, 2012) in R (version 2.15.2, R Development Core Team, 2012) using restricted likelihood estimates (REML). The ‘anova’ function in lme4 was used to determine the quality of fit between models using the AIC (Akaike’s Information Criterion). The pvals.fnc function in the LanguageR package ([37], version 1.4, 2011) was used to compute Markov Chain Monte Carlo (MCMC) p-values. Although this R function generates a p-value based on the t-statistic and MCMC procedures, only the MCMC p-value is reported here. The model fit was visually inspected using normal probability and residual plots.

### Ethics Statement

This study was approved by the University of New England’s Institutional Animal Care and Use Committee (Protocol Number: UNE-20111121THOMA). All animal husbandry and veterinary care, including blood draws, lighting regiments, swimming time, food preparation and individual animal room assignments were part of the normal care by the husbandry staff at MARC, in accordance with their National Marine Fisheries Service stranding agreement. Dive tags were designed to be small, lightweight, and attached using the same glue (NASCO tag cement) that is already used to attach identification tags to seals and other wildlife. The tags were easily removed during periods of illness or discomfort by cutting the hair beneath the tag.

## Results

### Hematology

**Fig 1:**
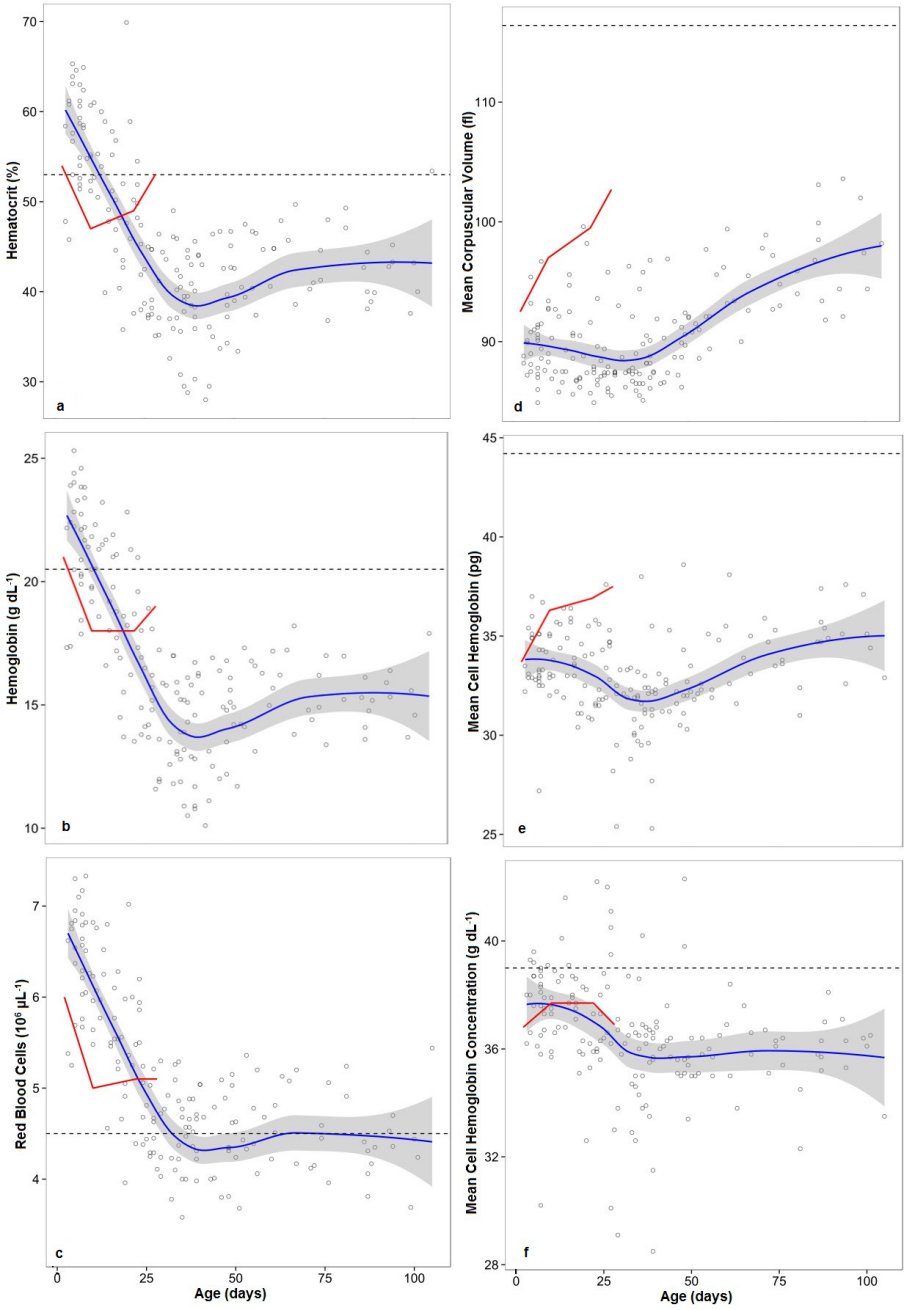
Effects of Age on Standard Blood Parameters in Rehabilitating Harbor Seal Pups. Effects of age on a. Hematocrit, b. Hemoglobin, c. Red Blood Cell Count, d. Mean Corpuscular Volume, e. Mean Corpuscular Hemoglobin and f. Mean Corpuscular Hemoglobin Concentration in rehabilitating harbor seal pups. The solid blue line represents the LOESS smoothing of the data from this study with a shaded area representing the 95% confidence interval. Each data point represents an individual blood sample (n = 173), from different pups (n = 21) in this study. The solid red line represents average data from wild nursing harbor seal pups from the literature [19], and the horizontal dashed line represents the average wild adult values from the literature [19].

Over the course of this study, 21 unweaned, but otherwise healthy harbor seal pups were admitted to the Marine Animal Rehabilitation and Conservation program and were sampled periodically throughout their stay (Table 2, age range: 2 days-105 days). All hematological parameters measured (Hct, Hb, RBC, MCV, MCH and MCHC) changed with individual age over the course of this study (Fig 1). Hct, Hb and RBC count were all elevated in the youngest pups, but decreased with age until approximately 30–40 days, at which time values remained unchanged throughout the remainder of the individual’s stay in rehabilitation. The pattern for RBC development is similar to that seen in wild harbor seal pups ([19]; Fig 1), though the number of RBC in rehabilitating pups reaches much lower values before leveling out. It should be noted that the lower values observed in rehabilitating pups correspond to the documented literature values for wild, adult harbor seal RBC ([19], Fig 1). The best-fit model of RBC, Hct and Hb in this study (S1 Table) reflects the decrease and leveling off trend observed, such that it includes a significant third-order polynomial effect of age (Age^3^ factor: p<0.001 for all).

MCV and MCH decreased slightly during the first 20–30 days of age and then increased throughout the rest of the animal’s rehabilitation. Neither MCV nor MCH reached adult values during the course of this study. The best fit linear mixed model for MCV and MCH included a significant third-order polynomial effect of age (Age^3^ factor, MCV, p<0.001, MCH, p<0.0001; Fig 1).

MCHC values decreased slightly for approximately the first 30 days of age, and then remained relatively unchanged. This pattern was similar to that seen in wild pups, and neither wild nor captive pups reached adult values (Fig 1) by the end of the respective studies. A second order polynomial effect of age was the best fit linear model for MCHC (Age^2^ factor, p<0.01). The effect of age on Hct, Hb, and MCV varied significantly between the sexes (Age x Sex interaction, Hct, p<0.05, Hb, p<0.01, MCV, p<0.01) such that males had consistently lower Hct, Hb and MCV values than females. It is unclear whether this is an effect of unequal numbers of males and females (14 females and 7 males) or a true effect and should be investigated further. Values did not significantly vary between the sexes in RBC (p = 0.0598) or MCH (p = 0.051) and was not a factor in the best fit model for MCHC.

### Diving

**Fig 2:**
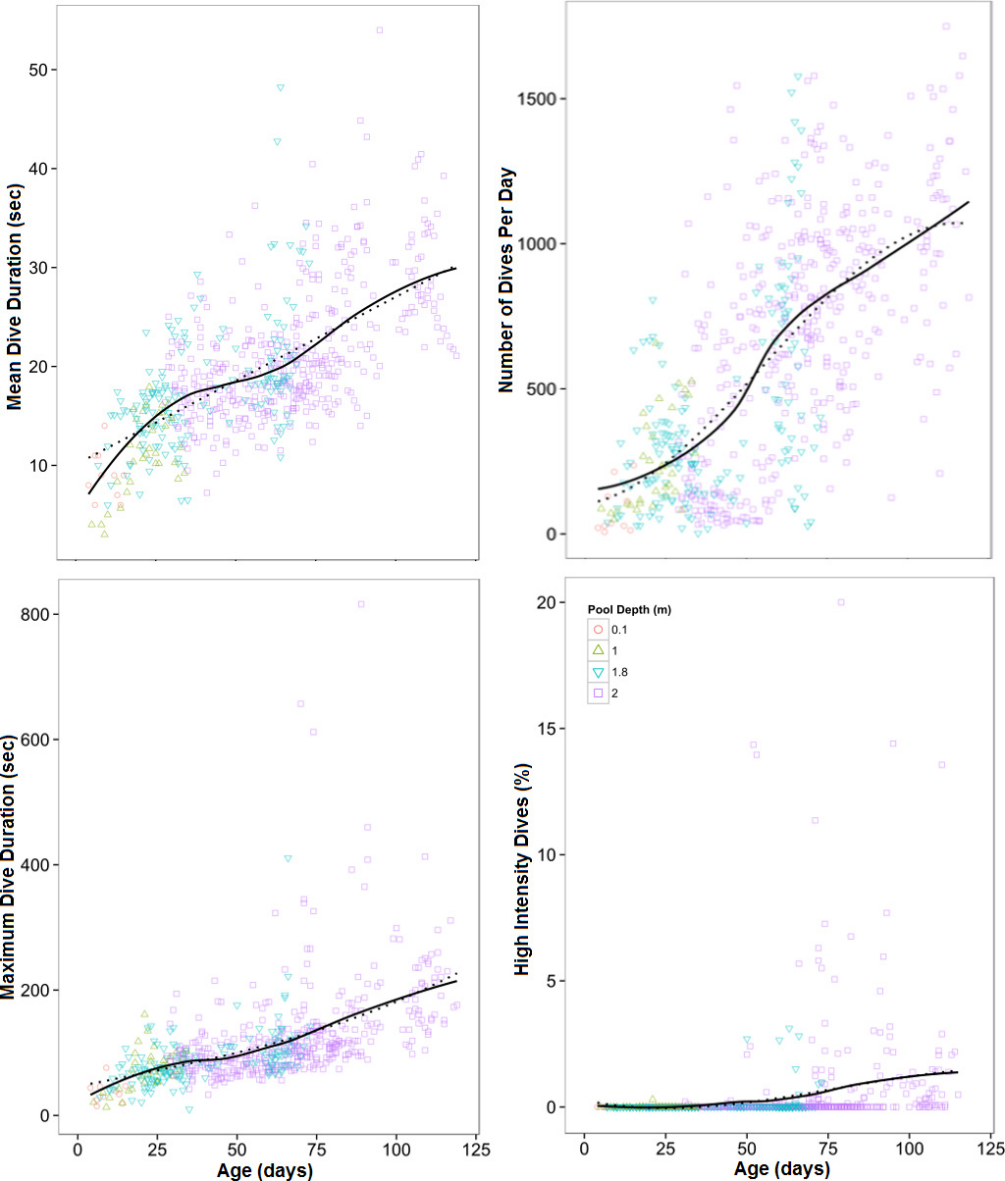
Effects of Age on Diving Behaviors in Rehabilitating Harbor Seal Pups. Effects of age on maximum dive duration, mean dive duration, number of dives per day and percentage of high intensity dives (dives > 150 seconds in duration). The solid line represents the LOESS smoothing of the data and the dotted line depicts a third-order polynomial effect of age (as suggested by the best fit models—see text for details). Shapes represent available pool depth (circles = 0.1 m, triangle = 1 m, upside-down triangle = 1.8 m, square = 2 m). Each data point represents an individual’s data for a given day (n = 610), for each pup (n = 15).

Surveillance videos were used to assess the duration of apneas in the shallow (0.1 m) pools of 7 harbor seal pups (ages: 4–20 days). A total of 14 pups (including all 7 that were videoed) were instrumented with Sensus Ultra Dive Tags. The data set included a total of 610 days of data collection, and 363,037 dives (ages: 9–119 days). Underwater breath-holds made by young harbor seal pups (<10 days old) were on average 7.9 ± 3.5 seconds long and did not involve any diving due to their shallow (0.1 m) pools. The maximum observed breath-hold made by a young pup was 46 seconds in duration. By the end of the study (110 days old), animals were capable of diving for an average of 22.45 ± 6.8 seconds; the longest two dives recorded in this study were 816 seconds (13.6 minutes) and 657 seconds (10.9 minutes) and were made by individuals that were approximately 89 and 70 days old, respectively. Throughout the course of this study, all diving parameters measured (Max DD, Mean DD, Num. Dives and HID) increased (Fig 2).

While all diving parameters increased with age (Fig 2), only mean dive duration (p<0.001) and HID (p<0.005) were significantly related to age (S2 Table). A significant positive relationship was also observed between mean and maximum dive durations with pool depth (p<0.05).

### Hematology and Diving

**Fig 3:**
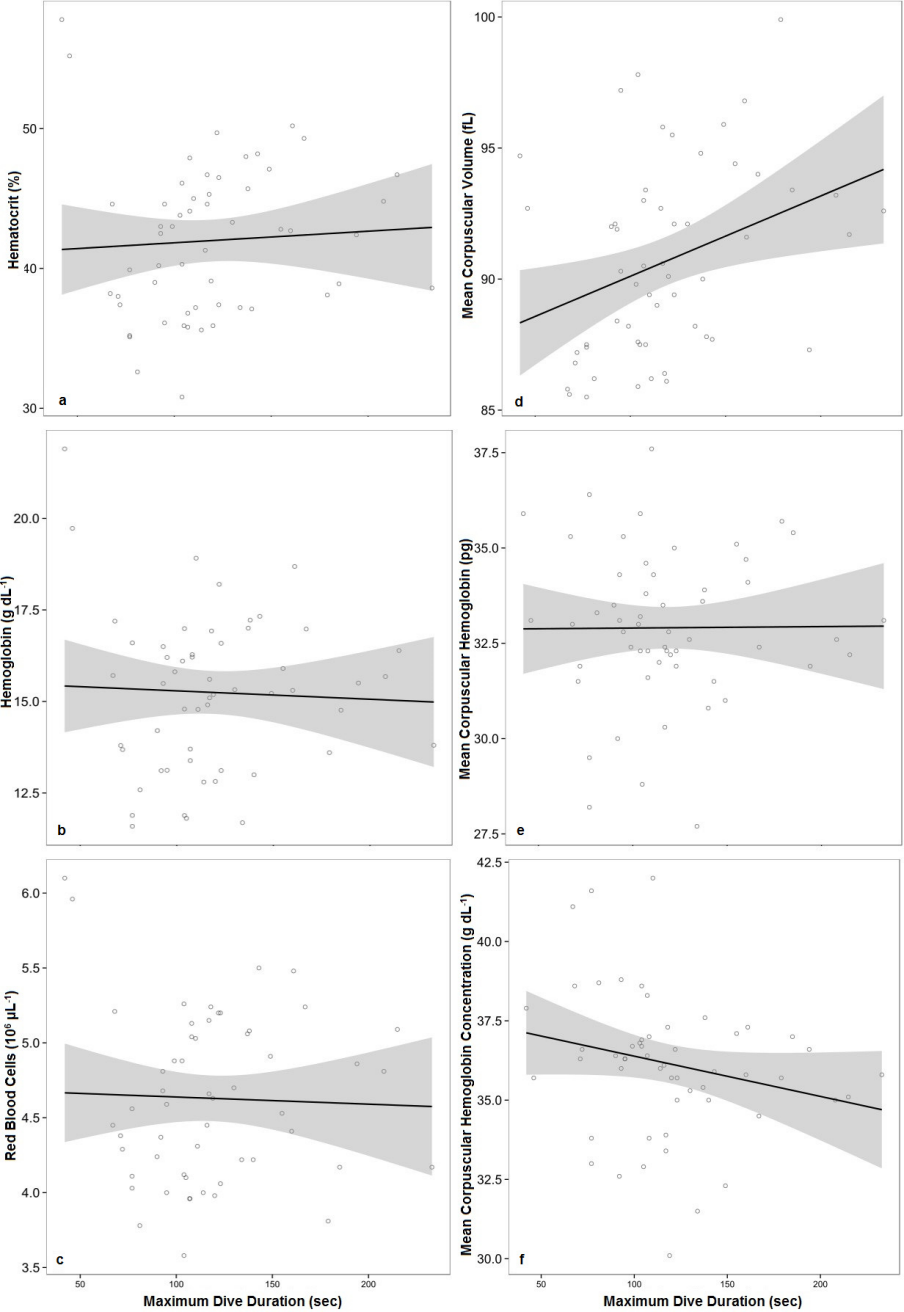
The Relationships Between Maximum Dive Duration and Standard Blood Measurements in Rehabilitating Harbor Seal Pups. Direct comparison between a behavioral diving parameter (maximum dive duration) and physiological parameters (Hct, Hb, RBC, MCV, MCH, MCHC). The black lines represent the lines of best fit, and the shaded areas represent the standard errors. Each individual data point corresponds to one measured instance from an individual.

Hematology measurements and diving parameters were directly compared to determine if the breath-hold experienced during rehabilitation was responsible for the development of blood oxygen stores. When compared directly to maximum dive duration (Fig 3), a correlation to any of the blood parameters was only observed for MCV (r(53): 0.34, p<0.05) but the r^2^ value for that positive relationship is 0.12. When blood parameters were compared directly to the percentage of HID, number of dives and pool depth, no correlation was observed.

To determine if a more complex relationship existed, the hematology measurements and diving parameters were directly compared in the same mixed model (S3 Table). The models suggest that Hct and MCV are significantly affected by HID (p<0.05) but the nature of that effect varied with age. Pool depth significantly affected MCHC (p<0.05) and RBC (p<0.05), such that MCHC decreased with an increase in depth and RBC decreased with age and pool depth. MCH and Hb were only significantly affected by age (p<0.05).

## Discussion

Rehabilitation of marine mammals has become a common practice in the United States, where facilities across the country have admitted an average of 1436 animals every year, with pinnipeds making up approximately 98% of those admitted [38]. As of 2004, facilities in New England alone admit an average of 172 pinnipeds annually. Though this rehabilitation is generally motivated by various conservation, animal welfare and scientific goals, monetary expenses, labor efforts and a lack of available validation of techniques and procedures makes marine mammal rehabilitation a controversial issue among scientists and stakeholders [38]. Some researchers investigating the effects of captivity on the development of young pinnipeds found a decrease in hematological values, which was assumed to be related to a lack of diving experience in captivity [26,27]. This study provides the first insight into the mechanistic relationship between hematology and diving experience during early-life captivity.

### Hematology

As had been noted in previous studies [26,27], hematological values for Hct, Hb and RBC were lower at the time of release than at the time of admission, but this study provides fine temporal scale details of this development that have not been previously studied in captive animals. In wild harbor seal pups, Hct, Hb and RBC all decrease shortly after birth and subsequently increase mid-way through the nursing period [19]. The initial decrease is thought to be caused by dilution due to the inability of blood cell production to keep pace with the rapid increase in plasma volume and body growth that occur while nursing on very fatty milk. Mid-way through the nursing period in wild harbor seal pups, RBC, Hb and Hct all begin to increase ([19] Fig 1) suggesting that blood oxygen stores begin to increase, possibly due to an increase in circulating Epo [19,39]. In this study, it appears that rehabilitating pups undergo the same general trend but this secondary increase does not occur until approximately 30–40 days of age, a full 20–30 days later than wild pups (Fig 1). At MARC, 40 days of age marks the average age that animals are weaned from gavage feeding to whole fish. However, this does not mark the transition from a diet of milk-replacement formula to a diet of fish, since animals are transitioned to a gavage-fed diet of homogenized fish at around 20 days of age. Because the change in diet does not correspond to the changes in RBC, Hb and Hct, it appears that the change in blood parameters is not due to diet. Similarly, no notable change in diving behavior was observed in this study at 40 days of age (Fig 2). Without an increase in diving-related breath-hold, there is no reason to deduce that an increase in Epo occurs at this time. It is unclear what is causing this rapid shift from decreasing Hb, Hct and RBC to an increase in these parameters, but it appears that the process may be more complex than shifting plasma volumes or increases in Epo.

In this study, animals were admitted to MARC with elevated Hct and Hb levels. This trend of elevated levels immediately after birth has been noted in several species of mammals (harbor seal: [15,19]; grey seals (*Halichoerus grypus*): [40]; Weddell seals (*Leptonychotes weddellii*): [41]; Steller sea lions (*Eumetopias jubatus*): [6], and humans: [42]) and is common due to a decrease in plasma volume. Such an effect could be intensified by dehydration and further loss of plasma water. As all of the pups in this study were maternally-dependent, and separated pups were generally observed alone for a period of 24 hours before admission to rehabilitation, it is likely that these animals were suffering from some degree of dehydration and would thus exhibit unusually high Hct and Hb values at the time of admission.

Further, pinnipeds store excess RBC in their spleen, which are released during diving, stress or sedation [43–46]; increasing circulating Hct and Hb. Abandonment, maternal separation, admission to rehabilitation, gavage feeding and handling several times a day at the beginning of rehabilitation could all lead to inflated Hct and Hb values in young pups. Throughout the course of this study, handling for gavage feeding and veterinary examinations may have become routine (i.e. less stressful), and therefore may result in a lesser amount of splenic contraction during blood draws, leading to decreased Hct and Hb values. As Hct and Hb values can be altered by varying degrees of hydration, and considering that MCHC (the mean concentration of hemoglobin in a volume of packed red blood cells) did not vary significantly throughout the course of this study, it is possible that hydration is a factor in early pup development. However, the animals in this study were fed to satiation three to five times a day, making dehydration an unlikely cause. Some studies have suggested that water movement within pinnipeds is complicated and not well understood, so the observed decrease in Hct and Hb could be affected by the complex shifting of body fluids [47–49].

An increase in the average size of the RBC (MCV) after approximately 40 days of age (Fig 1D), in conjunction with an increase in mean cell hemoglobin (MCH, 1E) and relatively stable MCHC and Hct (1A, F), suggests that although the concentrations of RBC were only slightly increasing throughout rehabilitation, Hb concentrations were increasing enough to sustain a constant concentration of Hb in a volume of packed RBC. Thus, after approximately 40 days, the pups in this study had a constant concentration of RBC, but these cells were slightly larger and contained slightly more Hb per cell than younger pups. This change in RBC physical characteristics suggests that blood oxygen stores did begin to increase slightly during rehabilitation. However, it should be noted that Hct, Hb, MCV, MCH and MCHC never increased to adult values during the study period, while these values have been reported to approach and even exceed adult values by weaning in wild pups (approximately 28 days of age, [19]). RBC was the only hematological value in this study that reached adult values (at approximately 30 days of age), suggesting that there were sufficient numbers of RBC present at that time, but they were either 1) diluted (leading to a decreased Hct) or 2) too small (leading to a decreased MCV) to approach adult oxygen storage capacity. In order for the number of circulating red blood cells in pups to have reached the number of adult RBC, and still exhibit a decrease in Hct and MCV, the erythrocytes must be physically smaller than adult cells and wild pup cells. These microcytic cells would thus be able to store less Hb (leading to the observed decrease in Hb concentration), and exhibit MCH values that are much lower than those recorded for adults and wild pups. Though the MCV increases during rehabilitation, it increases at a much slower rate than those in the wild, leading to rehabilitated animals with microcytosis and a theoretically decreased ability to store oxygen.

Theoretical blood oxygen stores can be calculated using previously described methods [6,50] and the following assumptions: 1) 33% of the blood volume is arterial and 66% are venous, 2) arterial Hb saturation is 95% at the beginning of a dive, but decreases to 20% by the dive’s completion, 3) all venous O_2_ is consumed during a dive, and 4) initial venous O_2_ levels are 5 mL dL^-1^ less than arterial O_2_ levels [2,17,41]. Since blood volume was not directly measured in this study, it can be estimated using body mass as 117 mL blood kg^-1^ body mass [51]. Given all of the aforementioned assumptions, blood O_2_ content of the youngest pups (<4 days of age) in this study was 27.0 ± 4.1 mL kg^-1^ as compared to the 32.8 ± 2.7 mL kg^-1^ for wild pups of the same age, as reported by Clark et al. [19]. By natural weaning age (28 days), blood O_2_ content of pups in this study had decreased to 16.7 ± 2.6 mL kg^-1^, while wild pups weaned with an O_2_ content of 27.0 ± 2.0 mL kg^-1^ [19]. Though these values are estimates, they further support our hypothesis that harbor seal pups in this study have a reduced ability to store oxygen in their blood, possibly due to the small size of their red blood cells.

Microcytosis can be caused by a variety of factors, though many are often linked to various forms of anemia [51–53]. Anemia is most commonly caused by a deficiency of iron in the blood, which would lead to a marked decrease in the amount of available hemoglobin. For canines, microcytosis is often related to iron deficiency anemia or anemia of inflammatory disease, which are generally caused by blood loss and persistent inflammatory diseases, respectively [51–55]. In this study, any blood samples that were collected during a period of illness (including both blood loss and inflammatory diseases) were removed from the study, limiting the possibility of having anemia-related anomalies in the blood data. Iron supplements (Mazuri Pinniped vitamin, One-A-Day Teen Advantage) and a herring-based diet [56] make iron-deficiency an unlikely cause for the observed microcytosis, though poor absorption remains a possible factor. It is also possible that microcytosis may indicate conditions other than anemia, as a recent study of microcytic canines found only 46.7% of the animals were actually anemic [57]. This evidence suggests that although the animals in this study appear to be microcytic, this condition is likely not caused by anemia or diet.

### Diving

Previous researchers have suggested that the observed low levels of hematological values in captive harbor seal pups could be caused by restricted access to diving. During hypoxia, erythropoietin stimulates an increase in RBC production [20,58] and prevents cell death of circulating RBC, [59,60] thus increasing the Hct. It had been assumed that without much space to dive, captive harbor seals rarely exposed themselves to long periods of hypoxia and thus the development of blood oxygen stores would be limited. Our study indicates that pools presented to captive animals (maximum of 2 m deep) provide enough space for young harbor seals to dive. The pups in this study were able to dive for longer periods of time, and were able to complete a higher proportion of HID (dives > 150 seconds in duration) per day as they aged. A similar trend was observed in wild harbor seal pups, [1,15,25] though wild pups were recorded to dive deeper and for longer periods of time at a younger age (8 min, age 1 week: [61]; approximately 9 minutes, age under 12 days:[25]; 8.5 min, age under 25 days: [1]; 3.6 min, under 25 days: [15]) than the captive pups in this study (maximum 13.6 min at 89 days, maximum under 25 days: 2.68 min). Harbor seal pup dive durations may be dependent on available depth in the water column [1,15,62,63] and pup motivation [63]. This study suggests that although the pool depth may affect the rate of development of these skills, it does not impede pups from performing long duration dives. Further, since their thawed herring diet generally floated at the surface of the water or was consumed before it reached the bottom of the pool, the pups in this study had little to no food-related motivation to dive and remain submerged. Most dives appeared to be for play and socialization purposes, though some individuals appeared to rest for short periods of time on the bottom of the pool (personal observations).

### Diving and Hematology

When directly comparing the hematology measurements and the diving parameters measured in this study, the data indicate that only MCV is positively correlated to maximum dive duration (Fig 3). Though the mechanism for this relationship cannot be inferred from this study, this correlation is of particular interest. As the erythrocytes of the study animals are thought to be smaller than those of their wild counterparts and this size difference is not thought to be diet or anemia related as previously mentioned, it is interesting to note that there may be an influence of extended breath-hold. One should not interpret these data to indicate causation, but this relationship should be further studied. This pattern has been seen before in nursing southern elephant seals (*Mirounga leonina*) where the onset of diving at approximately 3 weeks of age occurred concurrently with an increase in MCV [64]. However, this pattern was not seen in the precocial, wild harbor seal pup, as these animals dove often and early in life [15], but displayed no significant increase in MCV [19].

When investigating more complex relationships by directly comparing the hematology measurements and the diving parameters measured using linear mixed effects models (S3 Table), the data from this study indicate that Hct, Hb, MCH and MCV are most affected by age (as a third-order polynomial) and HID. This suggests that as pups age, those that perform more HID generally also have higher Hct, Hb, MCH and MCV values. Unlike previous studies, which indicate that depth drives dive duration, hypoxia [1,15,62,63] and subsequently the production of more, large RBC, [20,58] this study indicates that hematological parameters may be driven by frequent periods of extended apnea without an increase in depth. Since these parameters were not significantly affected by maximum dive duration or mean dive duration in the models, it can be inferred that the frequency of long-duration dives as opposed to depth, may drive this process in captivity. Further, the only parameter measured that affected HID in the model was age, suggesting that increasing pool depth has no effect on HID. Alternatively, RBC decreased with pool depth and age. This pattern may simply be indicating that younger pups are in shallow pools, and have high quantities of RBC (as discussed previously), though the interactive effect of the variables as indicated in this model suggests that a true interaction between these variables may exist. Similarly, MCHC decreases slightly with pool depth, which again, may be a result of young pups inhabiting shallow pools and having increased MCHC values or may be a true effect of pool depth. A wider range of pool depths and pup ages would likely be needed to fully distinguish the effects these two variables have on blood parameters.

## Conclusions

Ultimately, this study suggests that harbor seal pups raised in captivity generate an adult-like number of RBC, but these cells are small, less concentrated and can store less hemoglobin than their wild counterparts and wild adults. It can thus be assumed that captive pups can store less oxygen in their blood than wild pups, which may have a negative impact on their short-term post-release foraging ability. By investigating the fine scale details of this development, one can more clearly compare the development of these stores in captive animals and wild animals to determine any true effects of captivity. Further, inadequate pool depth or decreased diving experience, which were long thought to be the causes of reduced oxygen storage capacity in rehabilitating harbor seal pups, do not appear to be a limiting factor in the development of blood oxygen stores or diving ability. Captive pups that were given a maximum of 2m of water in which to dive were still capable of remaining underwater as long as their wild counterparts. This during-captivity development may help to explain how animals are capable of diving for long periods of time immediately after release [28,65]. Hematological development may be slowed in captive pups and the microcytic properties may be related to the percentage of long-duration dives made by an individual, but the mechanism of this relationship remains unclear. The data collected during this study will be useful to rehabilitation centers worldwide, as well as those studying the complex relationship between behavior and physiology.

## Supporting Information

S1 Table Summary of linear mixed-effects models used to describe the relationship between Age, Sex and hematology parameters (Hct, Hb, RBC, MCV, MCHC). Random effects represents a model run only taking into consideration the random effect of individual. Values represent the degrees of freedom (df) of the model and Akaike’s Information Criterion (AIC). * indicates the best fit model based on AIC selection process.
(PDF)

S2 Table Summary of linear mixed effects models used to describe relationships between diving parameters and age, sex and pool depth. Summary of linear mixed-effects models used to describe the relationship between Age, Sex and Pool depth on diving parameters (MaxDD = Maximum Dive duration (sec), MeanDT = Mean Dive duration (sec), Num. of Dives = Number of Dives performed in a day, HID = Percentage of High Intensity (>150 seconds in duration) performed during a day). Random effects represents a model run only taking into consideration the random effect of individual. Values represent the degrees of freedom (df) of the model and Akaike’s Information Criterion (AIC). *indicates the best fit model based on AIC selection process. Interaction Effect models and additive polynomial models were performed but are not shown.
(PDF)

S3 Table Summary of linear mixed effects models describing relationship between diving and hematology parameters. Summary of linear mixed-effects models used to describe the relationship between Age, Sex, Pool Depth and Diving Parameters (Maximum Dive Duration and Percentage of High Intensity Dives (>150 seconds) made in a day) and hematology parameters. Random effects represents a model run only taking into consideration the random effect of individual. Values represent the degrees of freedom (df) of the model and Akaike’s Information Criterion (AIC). *indicates the best fit model based on AIC selection process. All additive, interaction Effect and additive polynomial models were performed but are not shown.
(PDF)
