# Supplementary material for: Diving Related Changes in the Blood Oxygen Stores of Rehabilitating Harbor Seal Pups (Phoca vitulina)
Source: PLoS One. 2015 Jun 10;10(6):e0128930. doi: 10.1371/journal.pone.0128930 (PMC4465541; doi:10.1371/journal.pone.0128930)
Supplement: S1 Table — Random effects represents a model run only taking into consideration the random effect of individual. Values represent the degrees of freedom (df) of the model and Akaike’s Information Criterion (AIC). * indicates the best fit model based on AIC selection process. (PDF) [file pone.0128930.s001.pdf]

Model	df	Hematology Parameters (AIC Shown) Hct	Hb	RBC	MCV	MCH	MCHC
Random Effects	3	1198.42	912.2	445.1	948.84	746.19	743.66
Age	4	1149.94	861.53	352.07	821.38	744.24	732.06
Sex	4	1196.64	911.03	441.97	949.36	748.05	743.54
Age+Sex	5	1147.61	859.81	347.42	823.11	746.17	731.69
Age*Sex	6	1148.31	860.02	348.41	764.85	745.47	733.63
Age+Age^2	5	1053.6	768.4	262.96	818.83	713.07	**725.94***
Age+Age^2+Age^3	6	1003.44	738.18	220.69	736.34	707.78	727.17
Age+Age^2+Age^3+Sex	7	1001.54	736.9	215.55	737.75	709.38	727.83
Age*Sex+Age^2+Age^3	8	**998.31***	**733.53***	**213.82***	**724.05***	**706.62***	729.67
