# Supplementary material for: Diving Related Changes in the Blood Oxygen Stores of Rehabilitating Harbor Seal Pups (Phoca vitulina)
Source: PLoS One. 2015 Jun 10;10(6):e0128930. doi: 10.1371/journal.pone.0128930 (PMC4465541; doi:10.1371/journal.pone.0128930)
Supplement: S2 Table — Summary of linear mixed-effects models used to describe the relationship between Age, Sex and Pool depth on diving parameters (MaxDD = Maximum Dive duration (sec), MeanDT = Mean Dive duration (sec), Num. of Dives = Number of Dives performed in a day, HID = Percentage of High Intensity (>150 seconds in duration) performed during a day). Random effects represents a model run only taking into consideration the random effect of individual. Values represent the degrees of freedom (df) of the model and Akaike’s Information Criterion (AIC). *indicates the best fit model based on AIC selection process. Interaction Effect models and additive polynomial models were performed but are not shown. (PDF) [file pone.0128930.s002.pdf]

Model	df	Diving Parameters (AIC Shown)			
		Max DD	Mean DD	Num. of Dives	HID
Random Effects	3	6912.6	3966.6	9020.4	2479.2
Age	4	6726.9	3705.1	8622.1	**2451.8***
Sex	4	6912.1	3967.6	9019.5	2481.1
Depth	4	6851.9	3861.5	8910.4	2475.9
Age+Sex	5	6728	3707.2	8624.2	2453.9
Age+Depth	5	6717.8	**3686.2***	8609	2453.6
Sex+Depth	5	6851	3861.7	8908.6	2477.7
Age+Age^2+Age^3+Depth	7	**6709.5***	3688.6	8626.7	2454.3
Age*Depth+Age^2+Age^3	8	6710.3	3689.5	**8606.6***	2455.5
