# Supplementary material for: Diving Related Changes in the Blood Oxygen Stores of Rehabilitating Harbor Seal Pups (Phoca vitulina)
Source: PLoS One. 2015 Jun 10;10(6):e0128930. doi: 10.1371/journal.pone.0128930 (PMC4465541; doi:10.1371/journal.pone.0128930)
Supplement: S3 Table — Summary of linear mixed-effects models used to describe the relationship between Age, Sex, Pool Depth and Diving Parameters (Maximum Dive Duration and Percentage of High Intensity Dives (>150 seconds) made in a day) and hematology parameters. Random effects represents a model run only taking into consideration the random effect of individual. Values represent the degrees of freedom (df) of the model and Akaike’s Information Criterion (AIC). *indicates the best fit model based on AIC selection process. All additive, interaction Effect and additive polynomial models were performed but are not shown. (PDF) [file pone.0128930.s003.pdf]

Model	df	**Hematology Parameters (AIC shown)** Hct	Hb	RBC	MCH	MCHC	MCV
Random Effects	3	342.69	242.57	91.355	237.21	252.47	302.8
Age	4	344.68	243.75	89.884	237	251.41	265.94
Sex	4	343.76	242.25	91.997	238.11	251.57	304.8
HID	4	338.91	241.13	91.678	233.50	250.51	292.90
Max DD	4	344.66	243.87	91.993	239.21	251.66	298
Depth	4	333.49	227	73.251	237.68	249.03	302.24
HID+Depth	5	329.15	225.43	73.742	233.41	**247.32***	293.20
Age+Age^2	5	342.98	238.52	88.917	231.37	250.13	264.04
Age+ Age^2+Age^3	6	320.43	218.88	71.486	228.7	251.45	247.7
Age+ Age^2+Age^3+HID	7	315.98	**217.11***	70.611	**225.91***	249.79	244.68
Age+ Age^2+Age^3+MaxDT	7	322.43	220.88	73.465	230.63	253.26	249.15
Age+ Age^2+Age^3+Depth	7	318.79	217.83	**68.392***	230.44	253.21	249
Age*HID+ Age^2+Age^3	8	**312.05***	218.37*	70.009	227.69	249.68	**238.88***
Age*MaxDT+ Age^2+Age^{3}3	8	322.76	221.67	73.864	232.47	255.25	250.65
Age*Depth+ Age^2+Age^3	8	320.55	218.82	70.387	228.17	249.12	247.7
